# A community-engaged model for promoting youth behavioral health equity: voices from Washington State’s Community Prevention and Wellness Initiative

**DOI:** 10.3389/fpubh.2025.1617135

**Published:** 2025-08-12

**Authors:** Heather F. Terral, Jordan Newburg, Konul Karimova, Brittany R. Cooper, Gitanjali Shrestha

**Affiliations:** IMPACT Lab, Human Development, Washington State University, Pullman, WA, United States

**Keywords:** health equity, community coalitions, community engagement, prevention, Community Prevention and Wellness Initiative

## Abstract

**Introduction:**

Preventing youth substance use is a public health priority and community coalitions show promise for reducing youth substance use. The Community Prevention and Wellness Initiative (CPWI) is an innovative community coalition model that uses data to direct prevention efforts and resources to the highest-need communities in Washington State to reduce youth behavioral health disparities. The Washington State Health Care Authority (HCA) leverages federal and state funding to support CPWI, which implements evidence-based programs in collaboration with community-based organizations and other partners. Despite the growing evidence supporting community coalitions’ utility in prevention and public health promotion, less is known regarding how they can advance health equity in their communities.

**Methods:**

In 2022, we collaborated with HCA to interview 16 CPWI Coalition Coordinators about how they integrate health equity into their work. The second and third authors conducted interviews, which were transcribed professionally. Using a consensus building approach, the team conducted a thematic analysis of the transcripts.

**Results:**

Three general themes emerged: (1) Context, (2) Facilitators, and (3) Barriers. Coalition context included knowledge about the community’s demographics and dynamics, community capacity and past coalition functioning, and history. Facilitators of health equity promotion were rooted in relationships and adapting processes to enable community engagement. Barriers to health equity work were most commonly systemic, such as lack of access to healthcare, or lack of community resources. In addition to these themes, Coalition Coordinators highlighted key support needs (e.g., increasing funding and training) and shared important lessons learned, emphasizing the importance of relationship-building, cultural humility, and community-centered approaches.

**Discussion:**

Health equity efforts vary by community, shaped by local contexts and resources. Strong partnerships, community engagement, and trust were critical facilitators, while their absence hindered progress. Despite challenges, CPWI coalitions demonstrate resilience and creativity in addressing barriers. CPWI demonstrates a strong case as an innovative community-engaged model for promoting youth behavioral health equity.

## Introduction

Adolescent substance use has negative developmental impacts with long-term consequences, making prevention a global public health priority (1). Healthy People 2030 set five objectives to reduce adolescent substance use and raise awareness, but progress has been limited. Two objectives have shown little change (SU-04, reducing proportion of adolescents who drank alcohol in past month; SU-09, reducing proportion of past month binge drinking of people under 21) and two have worsened (SU-05, reducing proportion of adolescents who used drugs in past month; SU-06, reduce proportion of adolescents who used marijuana in the past month) (2). Adolescence is a sensitive developmental period, and stressors like global pandemics can heighten vulnerability to negative coping behaviors such as substance use (3). Despite the mental health challenges linked to COVID-19, youth substance use has decreased over the past decade and prevalence remains lower than pre-pandemic rates (4). Community coalitions are commonly used in public health as place-based interventions, leveraging multi-sector networks to address complex risk and protective factors (5). Their multi-layered, cross-sector structure enables the implementation of multiple strategies to address the complexity of risk and protective factors at the community level, supporting population-level impact (5). Although evidence supporting coalition models in general is mixed, randomized controlled trials of the University of Washington’s Communities That Care coalition model have shown success in reducing adolescent alcohol and drug use in the U. S. and Australia (6–8). However, less is known regarding how community coalitions can advance health equity in their communities (9).

Empowering and engaging community partners is essential to advancing health equity (10). Health equity means ensuring that all individuals have the resources and opportunities needed to reach their full health potential (11). It reflects a reality where differences in health outcomes are not disparately and disproportionately distributed among more socio-politically marginalized groups of people. Social determinants of health are systemic and environmental conditions such as economic stability, education, the built environment, and structural racism, and these determinants are key factors shaping equity and disparities (12). In community coalition work, health equity can manifest community-driven collective action with outcomes such as reduced substance use risk factors for vulnerable populations, increased access to green spaces for physical activity, or modifications to local ordinances to reduce exposure to addictive products. Health inequities lead to unjust and preventable burdens of disability and death, often resulting from poor social determinants of health, and remain a persistent challenge despite decades of public health efforts (13). Community coalitions may help address health disparities through their multi-level approaches as equity requires diverse, coordinated efforts (13–15). Several initiatives have aimed to support coalitions by intentionally embedding equity into their structures and processes. For instance, Wolff et al. (16) developed six principles under the Collaborating for Equity and Justice approach to guide coalitions in centering equity; these principles focus on community engagement and expertise, making injustice and its structural supports explicit, and fostering inclusion and community empowerment. Many coalitions have applied these principles (17, 18), demonstrating their relevance and value in guiding equity-centered coalition efforts.

Evidence suggests coalition-driven interventions can improve health outcomes for marginalized groups by connecting health and human service providers with communities (19). Coalitions also offer potential for increasing equity for underrepresented groups in research through engaged partnerships (20), yet health equity is rarely the primary focus of coalition evaluations (21). To address this gap, evaluators at Washington State University (WSU) partnered with the Washington State Health Care Authority (HCA) Division of Behavioral Health and Recovery (DBHR) to plan and implement a multi-method health equity evaluation. This paper highlights one objective: learning from Coalition Coordinators about their health equity efforts, including facilitators, barriers, and factors in coalition planning and implementation, as a foundational step toward understanding how coalitions define and pursue health equity in practice.

## Methods and results

### Program description

The Community Prevention and Wellness Initiative (CPWI) is an innovative adaptation of the Communities That Care model, using data-informed community coalitions to address youth behavioral health disparities in high-need areas (22). Launched in 2011 with 18 communities, CPWI has grown to 96 high-need communities across Washington State. It emphasizes shared leadership between communities and state-level partners while fostering local engagement through coalition building. CPWI coalitions receive funding, training, and technical assistance to implement prevention programs with community partners. Coalition Coordinators lead the strategic planning process using the Substance Abuse and Mental Health Services Administration’s Strategic Prevention Framework (SPF) in alignment with the Collective Impact Framework (22). This framework centers equity and includes five components: (1) a common agenda for problem-solving, (2) shared measurement for tracking progress, (3) reinforcing activities for collective impact, (4) continuous communication to build trust, and (5) a core coordination team (23). Coordinators receive equity training from various sources including but not limited to DBHR’s SPF OWL eLearning series, which is a six-hour training that weaves cultural competency throughout all sessions and features a dedicated session on the topic.

### Evaluation context, frameworks, and questions

Washington State’s 39 counties span diverse regions, from coastal communities in the west to high desert areas in the east, with a variety of agricultural communities in between. Cultural differences across the state are often described as the “Cascade Curtain,” reflecting a divide between urban western and rural eastern areas (24). What works in Seattle may not suit Yakima or Spokane, highlighting the importance of locally driven efforts. A key strength of the CPWI model is its community-driven approach, where each coalition develops a strategic plan tailored to local needs and data. Similarly, approaches to promoting health equity will vary by community. This study used qualitative methods to explore how CPWI coalitions across Washington integrate and promote health equity in their prevention efforts. The study was deemed exempt by the Washington State Institutional Review Board.

Designing the evaluation was an iterative process guided by elements of the Culturally Responsive and Equitable Evaluation (CREE) approach (25). CREE centers evaluation activities within a cultural context and history and calls for acknowledging, addressing, and integrating cultural and racial complexities, beliefs, and experiences into the evaluation process (25, 26). Following the principles of CREE, the evaluation team conceptualized and carried out the evaluation plan with cultural humility: reflecting on their own positionality and biases while building their knowledge on equity and the specific context of Washington communities. While we did not apply other theoretical frameworks in a formal manner, our evaluation design and analysis were informed by key concepts from implementation science. Specifically, our understanding of the equity-expanded RE-AIM framework (27) and the Consolidated Framework for Implementation Research (CFIR) (28, 29) helped inform the evaluation questions (listed below) and interview protocol.

- What is happening in CPWI communities regarding health equity work?
- What are the facilitators of health equity promotion?
- What are the barriers for health equity promotion?
- How can the communities be supported by DBHR?

### Interview protocol and processes

The evaluation team (except for the first author, all authors were involved in the original evaluation; see Author Contributions and Acknowledgements for details) collaboratively developed the semi-structured interview protocol, shared it with the DBHR team for feedback, and finalized it following multiple rounds of revision. The final script included questions on community context, Coalition Coordinators’ knowledge of health equity and social determinants, and coalition functions (see Supplementary material 1.1). To prepare, the second and third authors (JN and KK) reviewed coalition strategic plans and data books, summarizing key information into templates. These summaries streamlined the interview scripts by reducing redundancy, ensuring relevance to evaluation questions, and allowing Coordinators to contextualize responses. This process also helped co-leads familiarize themselves with coalition contexts, fostering trust and rapport while respecting Coordinators’ time. Following the first interview, minor revisions were made to some questions, and the team debriefed on notetaking, probing questions, and follow-ups via email.

### Participant recruitment

In May 2022, DBHR announced the study to CPWI Coordinators of 96 communities via online newsletter and contract managers. Coordinators indicated interest by June 15, after which DBHR provided contact information for 18 interested Coordinators. JN coordinated scheduling via email. Sixteen Coordinators participated in the interviews between June 22 and July 14, 2022. Coordinators received informed consent materials and interview questions in advance. They were compensated with $30 digital gift cards via TangoCard.com.

### Data collection

Sixteen interviews were conducted in English over Zoom, lasting 45–60 min. Participants could opt out of recording, and 16 transcripts were generated. Participants were assured of anonymity, and recordings were deleted after analysis. JN and KK alternated roles as an interviewer and note-taker. Having two team members per interview facilitated data familiarity and provided a backup for recording issues. Transcriptions by an external service were validated and corrected during coding as needed. All data were anonymized in the final report submitted to the DBHR.

### Data analysis

The team adapted the consensus-building approach proposed by Hemmler et al. (30) and developed a ten-step protocol for team-based thematic analysis (see Supplementary material 1.2). This approach accelerated analysis through teamwork and focused coding without compromising integrity (30, 31). We integrated both inductive and deductive approaches to the data analysis protocol by applying both open-coding and referring to the evaluation questions to identify themes. The team used Dedoose for coding. The entire coding process took approximately 12 weeks. First, JN and KK verified and organized the interviews to ensure consistent formatting according to the established template, including necessary metadata (e.g., interview date, participant and interviewer names, transcript verification date). They then independently open-coded the same five transcripts by taking notes to develop initial codes. To ensure diverse representation, the transcripts selected for open coding reflected all known contexts, including rural and urban, and newer and more established CPWI communities. Familiarity with the data from the collection process facilitated initial open coding and further analysis.

After finalizing the note-taking step, the entire team met, discussed the initial codebook by reviewing the descriptions and examples for each code, and how the codes might shape transcript interpretation. The team remained open-minded and flexible to capture key insights and avoid missing critical information. As a next step, JN and KK individually pilot coded one transcript per person, then swapped the coded transcripts and reviewed them separately and met to discuss any discrepancies in codes and came to a consensus on those codes. JN and KK then divided the remaining transcripts and met with other team members regularly to discuss coding progress and emerging themes. Throughout the process, JN and KK used an “Unsure” code to mark excerpts needing further discussion and met to achieve consistency in coding. During these meetings final decisions were documented, and the codebook and previously coded transcripts were updated and re-coded as needed. In the final stage, JN and KK divided excerpts by code and summarized each code in a separate document. Given the project’s timeline, the team’s expertise, and the evaluation purpose, each coded excerpt was assigned a single code for final summation. We prioritized the primary meanings of excerpts to identify key findings and how they aligned for generalizable insights. This approach balanced understanding specific facilitators and challenges of equity work with identifying overlapping patterns across communities. The final codes are available in Table 1.

**Table 1:** Codebook list of codes and descriptions or examples.

Level^a^			Code name	Code description and/or examples
1			Coordinator characteristics	Characteristics or experiences shared by the coordinator about themselves
	1a		Health equity practical knowledge/experience/skills	(Ex) Experience as CPWI coordinator (Ex) Experience as a public health worker or coordinator in another position (Ex) Whether they applied the terms (“What do these mean to you: health equity, health disparity, social determinants of health”) to practical work or examples; code their answer overall as well.
	1b		Positionality	(Ex) Acknowledging race/ethnicity, multilingual or not, previous personal experience as it relates to work (e.g., one coordinator shared being intentional about pronoun use because of a military background)
	1c		Barriers (Coordinator)	Examples/times a coordinator characteristic or experience was clearly discussed as a barrier. (Ex) When coordinators have to translate materials and they aren’t multilingual. (Ex) Coordinators sharing feelings of burnout/stretched out or doing more than the position requires.
	1d		Facilitators (Coordinator)	Examples/times a coordinator characteristic or experience was clearly discussed as a facilitator. (Ex) A coordinator being new--that itself could be neutral, but if discussed as an opportunity to build new relationships that have helped it’s a facilitator. (Ex) A coordinator having experience as an “other/outsider” helping them be intentional about creating space in coalition work. (Ex) Coordinators demonstrating/applying their skills and experience to facilitate an aspect of CPWI work (e.g., educating partners facilitates engagement; framing conversations/discussions to engage community members) (Ex) Coordinators advocating for something beneficial to their community (Lots of “I” statements used)
2			Coalition	Umbrella code for logistics, administration, and functioning of CPWI coalitions
	2a		Requirements	Related to CPWI coalition requirements and planning for membership and functioning. Also code examples/factors/descriptions that are neutral--not clearly a barrier or facilitator--either here or as “unsure” for discussion.
		2a.i	Barriers (Requirements)	(Ex) Lack of data on specific subgroups “hiding” inequities and impacts (e.g., LGBTQIA+ youth in small communities) [relates to HYS and other data from DBHR that communities are expected to use] (Ex) Issues collecting data DBHR requests or requires (e.g., collecting personal information as a deterring participation; difficulties administering surveys to groups) (Ex) Strategic plan - lacking guidance on creating plans; lacking guidance using/applying plans
		2a.ii	Facilitators (Requirements)	(Ex) Coordinators and/or coalition members working within or adapting CPWI requirements to improve program delivery and/or community engagement.
	2b		Programs/Materials	Excerpts related to program implementation and adaptation (e.g., adapting delivery or translating materials). Also pertains to related materials not specific to a program (e.g., informational or promotional material).
		2b.i	Barriers (Program/Materials)	(Ex) Issues translating and/or disseminating program and/or other materials - information about community resources; radio and internet PSAs; awareness raising/promotional materials (Ex) Lacking enough information/data to implement a program/implement well.
		2b.ii	Facilitators (Programs/Materials)	(Ex) Coordinator adapts delivery of information and/or surveys to engage subgroups that would otherwise not be included (or included equitably). (e.g., Keeping surveys in paper format, not just QR codes, for older adults) (Ex) Coordinators and/or coalition members adjusting the timing/format/etc. of programs to improve implementation and/or reach.
	2c		Membership (Diversity, composition, and recruitment)	Descriptions, examples, etc. relating to coalition membership, recruitment, and engagement.
		2c.i	Barriers (Membership)	(Ex) Difficulties with outreach or engagement of subgroups
		2c.ii	Facilitators (Membership)	(Ex) Active participation from subgroups (Ex) Ways coordinators or coalitions have/will respond to or otherwise address barriers they discuss (e.g., moving meeting times; having interpreters)
	2d		Internal communication and decision-making	Descriptions, examples, etc. of communication modes/methods (within the coalition) and decision-making processes within coalitions. Also code examples/factors/descriptions that are neutral--not clearly a barrier or facilitator--either here or as “unsure.”
		2d.i	Barriers (Int communication)	[Examples needed]
		2d.ii	Facilitators (Int communication)	(Ex) Examples or discussion about bringing information to coalition members and work groups to ensure decision-making is informed and subgroups are engaged.
	2e		External communication and decision-making	Descriptions, examples, etc. of communication modes/methods with community members, partners, etc. Also code examples/factors/descriptions that are neutral--not clearly a barrier or facilitator--either here or as “unsure.”
		2e.i	Barriers (Ext communication)	[Examples needed]
		2e.ii	Facilitators (Ext communication)	[Examples needed]
	2f		Partnerships	Descriptions, examples, etc. of partnerships between CPWI coalitions and other entities (usually but not always local schools or healthcare settings).
		2f.i	Barriers (Partnerships)	(Ex) Difficulty reaching out to organizations (no response, lack of organizations, etc.)
		2f.ii	Facilitator (Partnerships)	(Ex) Leveraging existing networks, sharing data, and co-planning events
	2 g		Meetings	Excerpts that describe logistics and processes pertaining to coalition meetings or events that occur within coalition meetings.
		2 g.i	Barriers (Meetings)	(Ex) Examples of difficulty engaging coalition members during meetings (e.g., people not giving feedback/input, some members always being the one to speak while others do not, difficulties having conversations due to linguistical barriers)
		2 g.ii	Facilitators (Meetings)	(Ex) Adjusting coalition meetings (e.g., offering multiple times or formats, changing meeting times to accommodate members, reframing conversations/prompting conversations) to improve engagement (Ex) Examples of actions taken by coalition members and/or coordinator to recruit coalition members, orient them to CPWI work, or otherwise get them involved in CPWI
	2 h		Formal and informal work groups	Descriptions/Examples of formal and informal work groups/committees and respective actions/work. Use this code only for excerpts relating to work groups within the coalition, not external work groups.
	2i		Barriers (Coalition, general)	Catch-all code; Excerpts that describe barriers within coalitions and/or their work not best captured in other coalition subcodes.
	2j		Facilitators (Coalition, general)	Catch-all code; Excerpts that describe facilitators within coalitions and/or their work not best captured in other Coalition subcodes.
3			Community	Characteristics and factors related to CPWI communities and contexts.
	3a		Description/Context	Descriptions or explanations of context from the interview (specific data points will be pulled from strategic plans). Also code examples/factors/descriptions that are neutral--not clearly a barrier or facilitator--either here or as “unsure.”
		3a.i	Rural/Urban	(Ex) Code information in the first question of the interview (summarizing Strategic Plan information) (Ex) Code excerpts that brought up that describes the urbanicity/rurality of a community.
		3a.ii	Race/Ethnicity composition	(Ex) Code information in the first question of the interview (summarizing Strategic Plan information) (Ex) Code excerpts that discuss the racial/ethnic composition of the community.
		3a.iii	Poverty levels	(Ex) Code information in the first question of the interview (summarizing Strategic Plan information) (Ex) Code excerpts that bring up the poverty/affluence of the community.
		3a.iv	Age composition	(Ex) Code information in the first question of the interview (summarizing Strategic Plan information) (Ex) Code excerpts that discuss age composition of community members
	3b		Barriers (Community)	(Ex) Attitudes/norms resistant to DEI work as a whole or for subgroups/topics (Ex) Lack of community capacity/resources (such as community funding, number of organizations, flexibility and availability of organizations and members, distance from other supports) or lack of readiness/openness (such as knowledge of DEI/equity, willingness to learn) (Ex) Difficulty engaging with community due to intergenerational or historical trauma, mistrust, fear of perceived authority, etc.
	3c		Facilitator (community)	(Ex) Attitudes/norms supportive of DEI work as a whole or for subgroups/topics (Ex) Networking and/or leveraging existing relationships for planning, sharing data, and work (Ex) Community capacity and openness (e.g., funding, number of organizations, flexibility and availability of members, knowledge of DEI/equity, willingness to learn) (Ex) Supportive community or sector leaders
4			DBHR support/actions	Actions or strategies DBHR might employ to support CPWI coalitions.
	4a		Training/guidance support	Note - put anything related to coalition members’ training here, whether it’s a need or description of a completed activity. (Ex) Training/assistance creating and using/applying strategic plans (Ex) How to have difficult conversations about equity, community development, and related topics in a health and productive way. (Ex) How to promote readiness (e.g., knowledge/education, willingness to engage) (Ex) Increasing accessibility of trainings (e.g., having trainings recorded, trainings available in sections/chunks, having a trainer in-person, having a trainer available during meetings)
	4b		Financial support	(Ex) Funding for participation - Incentivizing participation in coalitions with families and youth for their efforts; removing practical barriers to participation (e.g., via gas cards, childcare) (Ex) Funding for food - Providing funding for food to be provided at meetings and events (discussion of food making events more welcoming and worthwhile to community members) (Ex) Funding technology and infrastructure - Examples/discussions of funding needed to devices/hardware and hardware delivery/dissemination (Ex) Funding disbursement - Examples/discussions of issues with funding being siloed (via sources, requirements, or both) and communities having to “braid” funding
	4c		Material support	(Ex) Creating materials or adapting materials for coordinators/communities (program materials or others) - Examples/discussions of coordinators having to translate materials themselves and/or find translators for hire or volunteer work (Ex) Addressing larger community needs (e.g., providing assistance to community members with bills, utilities, etc. as participation in programming is less likely until basic needs are met)
	4d		Other requirements/supports	(Ex) Changing reporting requirements (e.g., making relationship building actions and networking a “deliverable,” making a clear list of actions that promote health equity, otherwise recognizing related work coordinators are doing) (Ex) Assistance networking with organizations (e.g., providing lists of organizations and areas they serve; providing guidance on reaching out to organizations, etc.) (Ex) Understanding “bigger picture” factors as they impact coordinator work (e.g., changes in legislature may translate to changes in what sectors can do, such as law enforcement responding to mental health crisis calls)
5			Success stories and lessons learned	These are success stories and lessons learned coordinators provide throughout the interview (not just in relation to those specific questions). These can be general lessons or success stories about coordinator work, specific lessons or stories related to CPWI work (e.g., program delivery), and so on.
6			Unsure/discuss	Code any segment that seems important but difficult to categorize here.

^a^Indicates the code’s level in an outline form. The left-, center-, and right-alignments in this column are intended to mimic outline style writing.

## Results

The results are organized around three key themes: (1) Context, (2) Facilitators, and (3) Barriers. These themes are further divided based on three parent codes: Coordinator-level, Coalition-level, and Community-level. This thematic structure, as shown in Table 2, aligns with our coding approach and provides a multi-level perspective on factors influencing, supporting, or hindering health equity efforts. Two parent codes, Support Needs and Lessons Learned, summarize insights and recommendations that emerged across the data. We share Coordinators’ Success Stories in full in Supplementary material 1.3.

**Table 2:** Visualization of organizing matrix of codes and themes.

	Theme^2^		
Parent codes^1^	Context	Facilitators	Barriers
**Coordinator** (*n* = 111)	**Subcodes**: Diverse professional backgrounds; varying readiness and confidence; influence of positionality	**Subcodes**: Personal commitment to equity and justice; advocacy skills; lived experience	**Subcodes**: Limited familiarity with the community; unclear role expectations; and insufficient structural support.
**Coalition** (*n* = 219)	**Subcodes**: Variation in structure, function, and maturity; impact of COVID-19 on coalition development	**Subcodes:** Flexible and culturally responsive approaches; youth empowerment; intentional relationship building; strategic partnerships	**Subcode**s: Rigid program requirements; limited cultural and linguistic adaptations; challenges with inclusive membership; insufficient community engagement
**Community** (*n* = 72)	**Subcodes**: Rural vs. urban communities; predominantly White, non-Hispanic populations; poverty and resource access as key equity concerns; political and cultural climate	**Subcodes**: General openness to prevention and willingness to support prevention efforts, especially when equity is framed to align with local values	**Subcodes**: Systemic and sociocultural factors

^1^The parent codes support needs (*n* = 72) and lessons learned (*n* = 40) are not listed as columns because these excerpts were summarized across the data rather than by themes (Context, facilitators, and barriers).

^2^As a reminder, these are listed as codes in Table 1 because they began as another code category, but it became clear these were more appropriate as themes.

### Context for health equity work

Coordinators have diverse professional backgrounds and varying levels of experience in prevention science, community engagement, and health equity. They reported varying levels of readiness and self-confidence when addressing equity-related issues. Positionality also played a substantial role in shaping their perspectives and approaches to equity work. One Coordinator reflected, “*It’s always present for me that if I do not make space for people that look like me, and have had experiences like me, those spaces will not exist.*” Coalitions varied in structure, function, and maturity. Some were long-standing coalitions with well-established leadership teams, while others were in early stages of development or rebuilding after disruptions such as from the COVID-19 pandemic. Communities implementing CPWI ranged from small, rural towns to more urbanized areas. Many communities were predominately White and non-Hispanic, though several communities were experiencing demographic shifts. Thus, in many communities, poverty and lack of access to resources were perceived as more pressing equity concerns than racial or ethnic disparities. In some rural communities, underlying distrust of government also influenced how CPWI efforts were received. One Coordinator described the challenge of doing health equity work in a politically conservative setting noting, “*I think they cringe and shudder and they do not like it, because it’s very foreign to them…‘Oh, that’s something Seattle has to worry about, that’s not something that we have to worry about.’…so when I do approach cultural competency with our coalition, as much as we can, I try to focus on class, poverty. I think people here want to do their best…but they do not agree with it politically.*”

### Facilitators of health equity work

Coordinator-level facilitators included values, advocacy skills, and experiences. Coordinators highlighted leveraging existing relationships and networks to build trust and foster community engagement. Coordinators’ personal commitment to equity and justice were also powerful facilitators. As one Coordinator shared, “*I see good in every child and I hate injustice…I think just caring, just being able to take the time to let go of reinforced and preliminary bias about different races and start over and look for the good in all young people.*” Another Coordinator described the systematic barriers they encountered and their determination to meet their community’s needs despite these barriers. They shared, “*you are shutting down a service or a resource… I’m stubborn and I realized that, you know they might not like it, but I’m still going to do it. So I still provide services and the language of the population we are serving.”*

Coalition-level facilitators included flexible and culturally responsive approaches, youth empowerment, intentional relationship building, and strategic partnerships. Coalitions adapted their work to meet the broader needs of community members. For example, they adjusted meeting times, offered hybrid or virtual meeting formats, translated materials into accessible languages and reading levels, changed program timelines and eligibility requirements, and provided surveys in accessible formats. One Coordinator added that in coalitions serving communities with a high proportion of seasonal and agricultural workers, schedules were further adapted to align with families’ availability, such as hosting meetings on Friday nights and launching programming in April before the busy season. Youth leadership was a powerful facilitator of equity work, both advancing equity goals and challenging adult members to reflect on their own assumptions. Coalition members’ visible and consistent presence in community spaces, along with their strategic partnerships helped them engage community members in culturally responsive and respectful ways.

Community-level facilitators included general openness to learning about prevention and a willingness to support related efforts. While community members may not always have the knowledge to connect prevention concepts with equity work, many were receptive when the ideas were framed to align with local priorities such as supporting the well-being of all youth instead of explicitly focusing on LGBTQ2IA+ youth. However, one Coordinator emphasized that a conservative community context does not necessarily prevent equity work and that coalitions need to have a nuanced understanding of local values and frame their messaging in ways that resonate with the community.

### Barriers of health equity work

Coordinator-level barriers included limited familiarity with the community, unclear role expectations, and insufficient structural support. Coordinators who were new to the coalition and the community often struggled with community outreach, planning, and implementation. High turnover of Coordinators and minimal transition support added to these challenges. Several Coordinators noted that their role was not well-defined, which resulted in misaligned expectations and added responsibilities that exceeded their designated work hours. This mismatch was especially noticeable in under-resourced communities where disparities exceeded their abilities and function.

Coalition-level barriers included rigid program requirements, limited cultural and linguistic adaptations, challenges with inclusive membership, and insufficient community engagement and partnerships. Requirements such as collecting personally identifiable information from program participants, especially undocumented immigrants, created discomfort and led to program dropouts. Coalitions struggled to access culturally and linguistically appropriate materials. Program materials were often only available in English or in translations that were not accessible to all community members. One Coordinator shared, “*…I do not even understand it [program material], and I’m a Spanish speaker too.*” While professional sectors were often well-represented in coalitions, it was harder to engage parents, youth (including LGBTQ2IA+ youth), racial and ethnic minoritized groups, and individuals from lower socioeconomic backgrounds. One Coordinator recounted a parent saying, “*I just do not feel comfortable speaking in front of these professionals, because I just feel like my voice is not valuable when I’m with these professionals in the room.*” Additionally, while professional members were often compensated for their time, community members were not, creating an equity gap in participation. Language barriers, lack of interpretation, and inaccessible materials further limited inclusive participation. Partnership barriers included resource limitations and siloed efforts.

Community-level barriers included systemic and sociocultural factors. Systemic barriers included poverty, limited access to healthcare, public transportation, internet connectivity, and recreational services. Coordinators shared that many community members, especially from marginalized groups, were in “survival mode” juggling multiple jobs and managing family responsibilities. This limited their ability to engage with coalition efforts. Sociocultural barriers were also prominent, especially in communities with limited readiness to engage in equity-related efforts. Coordinators described resistance to topics such as LGBTQ2IA+ inclusion, youth mental health, and substance use, especially in communities with more conservative political or religious values. Coordinators emphasized that community resistance was more likely when communities lacked ownership over programming, or felt disconnected from how the prevention message was framed.

### Support needs

Coordinators identified several areas where additional support from DBHR (the state agency funders and technical assistance providers) would strengthen their work including training, financial flexibility, material resources, and peer learning. While many Coordinators appreciated existing training opportunities, they expressed a need for more targeted trainings on topics such as systemic and cultural racism, LGBTQ2IA+ support, cultural competency, and health equity, especially in rural communities. Coordinators emphasized the need for training in multiple languages, flexible formats, and the ability to embed training within coalition meetings to increase engagement. Coordinators highlighted that the current funding structure often limits the ability to compensate community members, provide food and refreshments, or offer meaningful incentives for youth such as professional development opportunities. They added the need for more flexible funding to support equity-specific work including dedicated funds for technology to expand access to prevention efforts. Additional support needs included culturally and linguistically appropriate program materials, along with multimedia resources for outreach. Coordinators also advocated for a bottom-up, community-driven approach to planning and implementation.

### Lessons learned

Coordinators shared a range of lessons learned, emphasizing the importance of relationship-building, cultural humility, and community-centered approaches. Many Coordinators discussed the need to slow down, build trust, and engage communities in respectful, inclusive, and locally responsive ways. Several Coordinators noted that language translation alone is insufficient, and that effective engagement requires deeper cultural understanding, collaboration with trusted partners, and attention to how programs are framed and implemented. Coordinators also highlighted the importance of showing up in person, listening more than speaking, and embracing discomfort and imperfection in cross-cultural work. Coordinators emphasized that equity work must be intentional and ongoing. Coordinators expressed their desire for more inclusive coalition spaces, broader community conversations about equity, and dedicated action plans reflecting the complexity and urgency of this work.

## Discussion

Health equity efforts vary by community and are shaped by local contexts and resources. Strong partnerships, community engagement, and trust were critical facilitators, while their absence hindered progress. Despite challenges, CPWI coalitions demonstrate resilience and creativity in addressing systemic and local barriers. Findings from the Coalition Coordinator interviews align with those from other coalition-based health equity studies (9, 15, 32) and add to the limited body of qualitative research in this area. Tailoring efforts to the local context can maximize coalition impact (5). For CPWI coalitions, local context encompasses Coordinator characteristics, including their positionality and knowledge of health equity. Similar to Chouinard et al. (32), definitions of health equity varied among Coordinators, but they aligned in key concepts. Conceptual foundations such as understanding equity principles, incorporating equity-specific language in planning, and translating this into implementation are crucial for building coalition capacity (14). This study demonstrates that reinforcing these foundations through adequate resources strengthens coalitions’ capacity for health equity promotion.

Community demographics are also essential contextual factors. What may be equitable for one community such as virtual meeting options may be inequitable for another where internet access is limited. Engaging community members to identify appropriate approaches is vital for promoting health equity. Across interviews, Coordinators identified community challenges, including food and housing insecurity, violence, and suicide. While these challenges can complicate recruitment, retention, and engagement, they also link CPWI with other coalitions addressing similar issues, fostering collaborative resource sharing to meet community needs. Some communities may not exhibit racial or ethnic diversity but may reflect diversity in other forms, such as class, gender, and sexuality. Similar to findings from the HEALing Communities Study, a community coalition model aimed at preventing opioid-related overdose deaths (9), most Coordinators did not perceive coalition membership as fully representative of their communities. However, they actively sought to increase representativeness through partnerships to engage diverse community members, a strategy that is central to equity work among community coalitions (32). Building and sustaining these partnerships requires time and resources but remains fundamental to collective action and impact, both of which are drivers of health equity capacity (14). Coalition-driven systems change requires sustained, long-term commitment (15).

Multiple facilitators for promoting health equity were identified by Coalition Coordinators. It is no surprise that partnerships were frequently mentioned, and this is consistent with other coalition health equity research (9). Coordinators shared that community-based organizations serving different populations (i.e., Asian and Pacific Islander, migrant communities, Native American) connected coalitions with leaders and groups they would not have had access to otherwise. Organizations and agencies such as fire departments, county health departments, school districts, hospitals, law enforcement, and social services supported CPWI planning, implementation, and distribution. Multi-sector involvement is a requirement for CPWI coalitions; however, the presence of law enforcement can introduce tensions for some communities, particularly for those with larger undocumented immigrant populations and at greater risk for police-involved violence. Eliminating barriers by providing transportation to meetings, snacks or meals and childcare are examples of support that enabled more community members to engage in coalition work, which is consistent with other coalition health equity literature (32, 33).

However, not all coalitions had sufficient funding to spend on food, and often relied on local partnerships and other grants to supplement their CPWI funds. At the time of data collection, the annual cap for food spending was $1,000 per coalition; in part because of Coordinators’ feedback, this limit was recently increased to $1,500. Additionally, most Coordinators referenced COVID-19 as a barrier. Although all coalitions had to adapt due to COVID-19, for some, this led to improved functioning and attendance while others struggled to maintain community connections. Many Coordinators shared that their coalition activities were still recovering from the national emergency phase of the COVID-19 pandemic. Although none directly linked the pandemic to local health disparities, it is documented that COVID-19 disproportionately affects marginalized racial and ethnic groups (34). Aspects of CPWI infrastructure, some of which stem from federal requirements, were identified as barriers to health equity work as well, including reporting requirements and materials. Having to collect personally identifiable information from community members receiving CPWI programming is an unfortunate equity issue. This may be especially salient now as the policy environment is increasingly hostile to immigrant communities (i.e., Executive Orders 14,159, 14,160, 14,161, 14,163, and 14,165) and mass deportations are underway. Undocumented immigrants experience many risk factors related to mental health and substance use, and this risk is increased when considering intersectional identities, such as transgender immigrants (35). Providing alternatives to identifiable data can support greater uptake among vulnerable populations. Although DBHR navigates complex requirements and regulations at agency, state, and federal levels, it is continually exploring alternatives to meet the needs of communities to serve all individuals, particularly those who are most vulnerable.

CPWI and Coalition Coordinators use various strategies to promote cultural responsiveness. For example, CPWI Community Surveys are offered in a variety of languages upon request, such as Russian, Swahili, and more. Yet the majority of CPWI programs, materials, campaigns are in English and typically written at a college reading level. When CPWI coalitions adapt or translate materials for cultural or linguistic accessibility, those efforts are typically shouldered by Coordinators or community members who are not professional translators. These efforts, while crucial, reduce the capacity of Coordinators to address other responsibilities. Along those lines, Coordinators also shared that turnover and limited time for training new staff impedes coalition work. Coordinators often serve as jacks-of-all-trades to meet contract requirements and implement CPWI, but many reported feeling overextended, stretched thin, and burnt out. Lastly, Coordinators shared community-related barriers to health equity work. These included systemic barriers, such as access to prevention services and healthcare in rural areas, but also important social-cultural barriers. Multiple Coordinators noted hostility toward LGBTQ2IA+ related issues, often rooted in local political and religious ideologies. For Coordinators working in rural areas, especially east of the “Cascade Curtain,” there can be a deep mistrust of the government and anything perceived to be associated with “the left” and Seattle and Olympia, where DBHR is based. As homophobic rhetoric and policies are being proliferated in the national policy environment (i.e., Executive Orders 14,201, 14,187, and 14,168), the stigma in these communities will likely grow and keep LGBTQ2IA+ youth at additional risk for not just substance misuse, but homelessness, interpersonal violence, and suicide (36, 37). Some Coordinators adapted health equity framing to emphasize benefits for “all” youth to navigate ideological resistance.

Coalition Coordinators alluded to potential systemic changes that could be made at the state and federal levels. First, increased funding for prevention and health promotion is always beneficial, but communities cannot hold their breath waiting for substantial funding increases. Additional funding and/or revised funding requirements would serve to expand the list of activities and resources that coalitions could pay for to facilitate their work. Increased funds could be used to enhance educational and professional development opportunities for coalition and community members and increase work efficiency. Secondly, expanding the types of relationship-building activities and events that can be billable to funders could empower Coordinators to rebalance their workloads and duties, while also opening avenues for evaluating relationship-building within the CPWI model. Third, while there are resources for prevention training in Washington State and at regional and national levels [e.g., The Athena Forum, Prevention 101; The Athena Forum (38), PTTC (39), and Society for Prevention Research (40)], not all of these resources are received as “beginner” friendly. Consistent training across coalitions can enhance their capacity for health equity work (9). Furthermore, materials for training, programs, recruitment, data collection, and so on require more than single-forward translations to be truly culturally responsive. That is, English-to-Spanish or English-to-Russian translations are less robust and accessible than materials originally developed in Spanish or Russian. Engaging community subject matter experts can facilitate cultural responsivity and ensure the inclusion of cultural knowledge that increases the validity of multi-lingual materials, and such community members should be remunerated for their expertise (41).

### Limitations

There are important limitations to consider for this study. First, not all active Coalition Coordinators volunteered to participate in the interviews. Hence, we are uncertain whether the perspectives and experiences shared reflect the views of Coordinators in other CPWI communities. Coordinators were not involved in the development or revision process of the interview tools. Furthermore, they were not invited to any co-interpretation and shared meaning making sessions, thus our interpretations were not verified by participants. These results are only one state’s example; the context outside of the Pacific Northwest is likely different. Future evaluations should prioritize an equity-focused approach to capture how coalition efforts vary across different geographic, cultural, and policy environments.

## Conclusion

Across Washington State, CPWI Coalition Coordinators are collaborating with their communities to promote youth behavioral health and seek to reduce inequities in their communities. Despite the diversity of regions and communities across the state, Coordinators experience similar and related barriers and bridges to health equity promotion: strong partnerships, community engagement, and trust were critical facilitators, while their absence hindered progress. CPWI coalitions demonstrate resilience and creativity in addressing barriers, many of which can be remedied with additional funding and training/technical assistance. Insights from community members implementing CPWI indicate it is a strong case as an innovative community-engaged model for promoting youth behavioral health equity.

## Data Availability

The raw data supporting the conclusions of this article will be made available by the authors, without undue reservation.
