# Supplementary material for: A community-engaged model for promoting youth behavioral health equity: voices from Washington State’s Community Prevention and Wellness Initiative
Source: Front Public Health. 2025 Aug 12;13:1617135. doi: 10.3389/fpubh.2025.1617135 (PMC12378976; doi:10.3389/fpubh.2025.1617135)
Supplement: Supplementary file 1 [file Table_1.DOCX]

Supplementary Material

# Supplementary Materials

## CPWI Coalition Coordinator Interviews: Script and Questions

**Interviewer**: [NAME]

**Date**: [DATE]

[Brief introduction: Name, position at WSU] We shared this information via email, but to recap, these interviews are being conducted to better understand where your coalition and community is at with health equity promotion. Broadly, we want to know more about what’s been done, what is working well, and where more support is needed.

We will report the information gathered from the interviews at the group level—you will not be individually named; however, when we share success stories, we will identify your coalition so that your coalition’s success can be celebrated and recognized.

We anticipate this interview will take about an hour. This interview will be recorded via Zoom, and these recordings will be deleted once we analyze the data. Recordings are stored on an institutional drive and are password protected. If you wish not to be recorded, please say so now. You can also ask that I pause the recording at any time. [*If they agree to be recorded, turn on the recorder*]

If, at any time, you share information you do not want to be shared with DHBR, please let us know. Also, if there are questions you would rather not answer, please feel free to say so and we’ll skip that question.

Do you have any questions before we proceed? If any come up, feel free to ask, or you can always email my supervisor [*Gitanjali Shrestha at* [*gshrestha@wsu.edu*](mailto:gshrestha@wsu.edu) *or Brittany Cooper at* [*brittany.cooper@wsu.edu*](mailto:brittany.cooper@wsu.edu)].

**Questions**

1. **We have had a chance to review your strategic plan and related documents and based on what we read it looks like [*summarize the work that the coalition is doing, and the community it serves. If the information on coalition work, interviewer can ask clarifying question.*]. Is there anything else you would like us to know about you and your coalition?**

- [NOTES]
  1. **Could you tell us more about your own experience as a coordinator?**
- [NOTES]

1. **We are interested in learning more about your coalition to help us better contextualize the information you provide. Having said that…**
   1. **To what extent does your coalition membership reflect the diversity in your community?**

- [Notes]
  1. **Could you describe how your coalition functions and communicates, including with partners and other stakeholders?**
     1. ***Prompt if needed*: For example, does your coalition have shared leadership and power, participatory decision-making, two-way open communication?**

[NOTES]

- 1. **Are there other structures and processes in place in your coalition to promote equity, diversity, and inclusion within the coalition membership?**
- [NOTES]

1. **We are interested in learning your perspectives about health equity, health disparities, and social determinants of health, and especially what these terms mean in the context of your work as a coalition.**
   1. **Let’s start with the term health equity – what do you think about when you hear this term?**

- [NOTES]
  1. **What do you think about when you hear “health disparities”?**
- [NOTES]
  1. **What do you think about when you hear “social determinants of health”?**
- [NOTES]
  1. **Do you use these terms when discussing your coalition work? If so, how do they apply?**
- [NOTES]

1. **As mentioned, we know that each Washington community is unique regarding health inequities and ways to address them. Can you describe what health inequities look like in your community?**

- [NOTES]
  1. **How would you describe the community’s response toward diversity, equity, and inclusion promotion?**
- [NOTES]
  1. ***Follow-up prompt, reword as appropriate:* To what extent is the community supportive of, resistant to, or apathetic towards working to advance diversity, equity, and inclusion in a meaningful way?**
- [NOTES]

1. ***Ask about the kinds of tasks conducted day-to-day if the Strategic Plan specifically names a workgroup:* Have committees and workgroups been established by the coalition to conduct tasks related to health equity promotion?**

- [NOTES]
  1. **Does the coalition participate in health equity workgroups or committees set up by others outside of your coalition?**
- [NOTES]

1. **[*Insert personalized statement: In your strategic plan, you have identified xx, xx as area of focus for health equity work*.] In general, how would you describe your coalition’s efforts to address these health equity issues identified in your strategic plan? What components from your strategic plan have you implemented?**

- [NOTES]
  1. **[*alternate question, if needed:* What are some ways in which your coalition has served underrepresented/ underserved groups in your community?]**
- [NOTES]

1. **Have you participated in trainings related to health equity or cultural competency?**

- [NOTES]
  1. **Trainings offered by DBHR?**
- [NOTES]
  1. **Trainings offered by other organizations? (*What were those organizations?*)**
- [NOTES]
  1. **Are there any remaining training needs you have in this area?**
- [NOTES]

1. **Based on your experiences, what factors or conditions support or hinder your ability to promote health equity in your community?**

- [NOTES]
  1. **(*Are some of these factors more salient at specific phases, for example*, in the assessment phase, in the planning phase etc.)?**
- [NOTES]
  1. **Do you think some of these factors are unique to your context?
     (*e.g.,* geographic region, population, affluence, racial/ethnic composition, age composition, partnerships)?**
- [NOTES]
  1. **[*If interviewee mentions DBHR requirement or EBP requirements, we will ask follow up-questions****:*
     1. Can you tell us more about that?
     2. Can you provide us with specific examples?
     3. Do you have suggestions for how to address that challenge?]
- [NOTES]

1. **Do you have any lessons learned related to promoting health equity in your community?**

- [NOTES]

1. **Are there specific strategies or tactics in which DBHR might better support coalitions in promoting health equity?**

- [NOTES]
  1. **[*If interviewee mentions, EBP requirements, strategic planning etc., acknowledge the response and ask if there are any other ways in which DBHR might be better support coalitions in promoting health equity?*]**
- [NOTES]

[*If there are any interesting or unexpected findings from the quantitative analysis, we plan to ask coalition coordinators for their input on interpreting the findings. These questions will be developed after quantitative analysis is completed*.]

**If we run out of time, ask – can we come back to you for success stories and future plans?**

1. **Are there any evaluation results or success stories you would like to share about your coalition and its efforts to promote health equity in your community?**

- [NOTES]
  1. **How, or in what ways, do you think your coalition’s work has most benefitted underrepresented/underserved groups in your community?**
- [NOTES]

1. **What are your future plans for promoting health equity in your community?**

- [NOTES]
  1. **Has this conversation sparked any ideas?**
- [NOTES]

1. **Is there anything else you would like to share?**

- [NOTES]

*Optional follow-up for questions that need more clarity* – **In reflection, I’d summarize what you’ve said [*Summarize*].** **Do you think that’s an accurate reflection? Did I miss anything?**

[NOTES]

**Thank you again for your time and participation! If you have any questions or follow-up thoughts, feel free to contact Gitanjali Shrestha at** [**gshrestha@wsu.edu**](mailto:gshrestha@wsu.edu) **or Brittany Cooper at** [**brittany.cooper@wsu.edu**](mailto:brittany.cooper@wsu.edu)**.**

## CPWI Qualitative Data Analysis Protocol

This protocol is written to guide the data collection and team-based thematic analysis of the CPWI Coalition Coordinator Interviews focusing on understanding the operationalization and promotion of health equity in the mentioned communities.

**Step 1. Clean, verify, and organize the data**

The research assistants will divide the transcripts and clean and verify interview transcriptions by listening to audio/video recordings as the interviews are conducted and ensure that all data is formatted similarly according to the Verified Interview Template, which includes an interview date, interviewer, and participant names, verifier name, and verification date. The original and verified transcripts will be retained. The research assistant will continue the verification of the transcripts as they follow the steps described below based on their availability.

**Step 2. Reviewing five transcripts for note taking**

After data cleaning and verification, each research assistant will individually read the same five interview transcripts selected from the interviews conducted. The five interview transcripts will be selected to be representative of urban and rural coalitions from both the east and west sides of the state from older and newer cohorts. In a separate document (i.e., memo), the research assistants will take notes on the patterns of data that stand out to them based on the research questions.

**Step 3: Develop a codebook**

After finalizing the note-taking step, the research assistants will meet with the rest of the team members to compare their notes/memos and create a Codebook in an Excel document based on consensus.

**Step 4. Import the Codebook to Dedoose**

**Step 5. Piloting the Codebook**

The research assistants will randomly pick two verified transcripts that were not used for note-taking before and individually code one of them based on the developed Codebook. Then, the research assistants will swap the coded transcripts and review them separately. The team members will meet after the coding to discuss any discrepancies in codes and come to a consensus on those codes. They will also make any needed changes to the codebook based on the pilot test.

**Step 6. Individual coding**

The team members will divide the transcripts and start the coding process based on the Codebook.

**Step 7. Conducting regular code meetings for “unsure” codes**

As the research assistants continue coding, they will code segments that they are unsure about or that do not fall under any code included in the Codebook as “unsure” in Dedoose and schedule regular coding meetings to review and make a decision on “unsure” codes.

**Step 8.** If, during the coding meetings, the team members add new codes to the Codebook, the team members will review the coded transcripts and retroactively code the previously coded files based on the new codes.

**Step 9.** The team members will meet and, based on the final codes and codebook, will decide on the themes and draft the first draft of the report. The team members can divide the themes among themselves and provide descriptions and quotes.

**Step 10.** After the finalization of the analysis process, the team members will meet and make necessary updates for this protocol for future analysis based on what worked well, what steps are redundant, etc.

## CPWI Coalition Coordinator Success Stories

- In facilitating *a prevention program*, one of our coalition members …. wanted to know how we can do this program [with] their students [who] are refugees and immigrants, and don't speak English at all. And a lot of them, as she explained, have suffered trauma. So, they invited us to come and do this program. We had two-week long sessions where our teaching artist worked with every class. So, we probably served at least 80 or 90 kids. And it was so cool because music is universal and they created their poetry, lyrics, their rap. So many people think that rap is bad. It's not all bad. There's good and there's bad. But anyway, they created in their own languages. They wrote poetry. There was one student that wrote this really long poem and he did it in his language. And our counselor told us that he was talking about how writing helps him overcome his trauma. So, I think that's something that I'm the most proud of. I think it's really cool that we were able to serve that many students and had such a great response.
- For one of our campaigns... which is the state youth marijuana prevention campaign… we bought like 700 school supplies and we're going to label them with positive messages that help educate kids about substance use and encourage them to focus on the positive things that they can do. Ultimately those school supplies are going to our local middle and high school kids that need school supplies. And that's something our coalition decided which was super great. It's a way that we need to spend this money and also support kids that really did need those supplies...I'm [also] excited that we're going to be in the pride tomorrow… We were able to buy some supplies and giveaway items that were imprinted… We're going to be there and have a presence and we also purchased a bunch of stickers that had some cute LGBTQ+ friendly messaging with rainbows and “be yourself” [messages]. And the kids love them and it's like kind of a subtle thing, but our hope is to really do as much as we can to support that community and then also make sure our stickers, graphics, imaging and messaging reflect the diversity of our community. And that hopefully it speaks to a broad range of people.
- I think there have been the partners have been willing to join in this work. Some of the key partners like the hospital and the school district and some of the local leaders, the police chief. So I think the fact that there is buy in and interest and engagement in some of these local leaders and organizations is really helpful and that will help the group move forward…I would say perhaps from what I've heard, the engagement level is a big pro and you don't necessarily get that in every rural community. So I am thankful for that.
- I think it's a success that we have a solid coalition, that we just formed… We wrote a strategic plan that was approved, and we have great community partnerships. We're partnering with the police and fire, the city, the school district, our mental health serving organizations locally. So, I'm proud of all of the relationships and partners that we've made….I will say we just visited our first evidence-based program that we're doing this summer.... We're serving 30 Native youth in our community, 13 -16-year-olds, and they meet every weekday for a month. They do a paddle out on the sound. And just seeing the kids... they are learning all of these woodworking skills, they have a culinary school, they're learning all this nature immersion stuff. It was just a really inspiring program….And offering this program for a full month that gives kids a place to go all day, every day during the week, and then also offering all these skills and pro-social opportunities, connecting them with mentors at the tribe, it was really cool.
- The biggest thing that comes to mind is [that] because we all went through a difficult challenging time together, I think we almost feel closer and more cohesive as a team because we did allow time for sharing. And we talked about struggles and concerns and hopes and aspirations. And I think some of those conversations were really meaningful and brought us together and perhaps even a tighter bond. I can say I love my coalition members. I want to spend time with them outside of meetings. I want to get to know them better. I feel like we're all in this together and we're working on a common purpose and it's worthy of our time. And I think we all like to be around like-minded people and so we have this common goal. And I think going through the challenging times made us even stronger as a team. And so now that we're coming out the other side and getting to meet more in-person, I feel like we have a really tight-knit grip that's ready to roll up their sleeves and start getting busy again, doing some good work.
- My success story is that I was able to partner with a very unconventional group. We're able to make it a community wide event, had great participation and feedback afterwards. And the goal is unity. And I feel like we're achieving that in [in our community]. I really do. That project helped launch more conversations. And again, following after that, probably a few months later, then you get a health equity policy in the school district because we are having those conversations. So, that to me is a success. And I think it all goes back down to, if I can help empower people, to find their voice and speak on behalf of themselves and their families and the communities they're representing, you get better solutions. You get better engagement. And I feel like everything's just better. It's a lot more successful.
- I gotta say we have gone from virtually never having a Spanish speaker meeting to making sure that we have interpreters every month, enough that people trust they're going to be there and then people show up, to having connections in the community, to having plans for how we're specifically going to reach out to the community and having voices in the coalition that as far as I know, we've never had before. And not only is that great and hopefully we'll be able to put our weight behind programs and policies and whatever is we know more that needs to be done, but… equity and bringing more voices to your table is not just to check a box. It genuinely makes such a difference, not only in what you hear, but it makes such a difference in energy, when you don't know what everyone's going to say and people are saying things you didn't know before. And so I do consider that to have been a success, that I have seen the coalition shift, I've seen their goal shift, and I hope that I've helped with some of that, but so much of that has been these incredible women who have been willing to put their time and their energy into making sure that their community has equitable access. So, you know, whatever happens with this, I do think that an incredible amount has been accomplished so far, I'm super proud of it.
- I have one success story where it was in 2020 when the Healthy Youth survey was canceled, we did a mini-Healthy Youth Survey with just our school. And all the questions had to be approved by the school board, and we had a question on there about gender, sexuality, gender identity, and those traditionally haven't been on our school survey because they're questions that they opt out of. And I went to the school board meeting with all kinds of paperwork and articles that I was going to share about how drug use, depression, mental health, and suicide all affect LGBTQ kids more. But the school board didn't even question it. They approved it without a blink, and so I was happy that we were able to get those questions approved...
- I think we kind of already talked a little bit about how we're going to run our family program, that's a big [success story]. And also our youth program, which we did a one trial run before our end of the school year and we're going to continue in the fall. Really, for us, it would be a making sure that people that are affected or the target population for the program or event or activity are involved in the planning process. And we make sure we understand what their needs are and what they think would work best to integrate that into our programming. I think that that, over everything, is our plan for making sure that we have representation from those that we're going to be serving.
- I think finishing our strategic plan was a big success being that I was told that I got a late start. I started at the end of October. I felt like the strategic planning process was very chaotic and rushed. I feel the process was quite a bit and trying to navigate that as a new coordinator and a community with members and partners that were also new to the process.  I think getting that finalized and approved was a huge success. I think we've done an educational workshop [Name]. I think that was a big success for the coalition. Got a lot of great feedback. I think it was one of our, I think it was a good way to kind of get our name out there and show what we're about and what we do, what we can do.
- Success stories would be my youth coalition. They're amazing. They've gone from... like I said three kids, and they finally busted out and we recruited a bunch of kiddos. I think what happened was we finally got to April, and I said ‘okay stop focusing on high schoolers, we're going to hit the eighth graders. Because they're going to be high schoolers next year and we're going to get them in the spring and keep them all summer long and then they'll be ninth graders.’ But in casting the net wide for eighth graders we ended up getting some other kids from the other high school that we hadn't even really talked to. And we ended up getting some more ninth graders that just heard about it and decided to show up. But what happened was then their advisor got sick and couldn't come on the day of the event and I did not step into the gap, the kids took it on, the three, and they lead. I was so proud of them, they did a great job of running circles and facilitating group conversations and I gave them stuff about cannabis to talk about, but they facilitated the group process. And considering that they've really only been meeting since December, I am super proud of the way they've come. And the fact that they've decided to do this YPAR thing and they're tenacious. They're not going to let me or us, the coalition ignore the importance of the equity pieces because that's absolutely what they care about. So I'm proud of them.
- Our biggest plus right now is partnering with other organizations that are already doing things. Whether because we're small, CPWI is really our only funding so…we're a nonprofit of our own making but we're small. If the youth and family services is doing a needs and resource assessment, I want to be part of that committee, or at least get the results and be able to use it, rather than try to reinvent that wheel and do it ourselves. And so I say, “sure I'll be on that committee, you know, and I might have some information that would be pertinent and relevant to their—whatever they're doing there.” So we can add to their knowledge base they might not be thinking of kids, well [Youth and Family Services] would be. Whatever it is, they might not be, it might not be right on their view, to take into account, you know young people. And what they're doing. So between [Place] youth and family services and the school district, and the domestic violence here [in place], they work with teens a lot and they have teen programs. We just try to add to that and partner with them, rather than you know silo and do our own thing.
- Okay, so, I had already mentioned [Program] which we do [for] 10 to 14 year-olds and their parents, and so we just started our second group now. I also do [Program] of [Place], which is a mentoring program and we utilize a lot of college students, which is really cool from [College]. They have a partnership to partner with youth in the community, so we go through a process—[not “we,”] I subcontract with them [to] match those mentors to mentees which has really been nice. Because we have kids and having the college students be able to be mentors throughout their school year has been really good. And it helps us because it's really hard to find mentors that are available for kids after school. I'm just entering the data in for our program and from January through May we had [more than 500] mentor match meetings. So I think that has been one of the strengths that we've been able to have the universities help with that.
- I think, to me the things that we're most proud of during the pandemic, especially we started a Women in recovery group, this was women who typically don't have homes, there were also survivors of domestic violence. There were no women only recovery groups in our community at the time and most of the groups were co-ed. A lot of the women didn't feel comfortable attending them, they were also in basements in the evenings of like churches and things like that, and so the women didn't feel safe attending and so we partnered with [Organizations with city name in them] to create a women's only recovery group. And this was for women at any stage of recovery. It wasn't like a typical AA where you had to be sober and abstinent from different substances. This was very much “come as you are”, and it was downtown on a bus line in a women's only facility and a well-lit area, in a well trafficked area so there's buildings and people coming and going all the time, so the women felt safe, and this was something that we provided with partnerships and with our funding.
- I mean, I think that our focus, I mean, this is what I heard from our last meeting, because we just had our June meeting because it was our planning meeting, but I think we've talked so much about how we want to empower youth and want to do more for youth. I think we are doing more for youth indirectly, but everyone is like, we want youth at the table. We want youth at the table and I was like, "Okay, y'all you have to work to make that possible." Sure. I have some school staff. We have a youth group that we do work with and we've engaged for, they've been a part of our prevention club for five years. It's actually really cute. We engaged them when they were in sixth or seventh grade and now they're 10th graders. They're going to be 11th graders. They're really great. That's our youth club.
- And that's something that we're trying to do here at coalition, as well, empower the parents, empower the community. Because their voice is very strong, as far as, ‘you're bringing a very, very strong point, you're a parent component to what we're doing. And what we are trying to do is reach parents like you, is trying to reach the youth that you have at home, so how do we do that? You are the key mover to what we're trying to do.’ So that's what we are trying to do. And the youth voices is really strong, because they go out and actually talk to their peers about what we are doing. It's better for them to get the information from a youth than me going to a classroom and saying, "Hey, substance abuse." And just kind of give them information about that. Or even with the [Name] campaign that we have, it's better for the student to disseminate that information to younger students, that way they can better understand what's going on. It's peer to peer information, rather than an adult trying to come and tell me what to do.
